# Dynamics of SNARE Assembly and Disassembly during Sperm Acrosomal Exocytosis

**DOI:** 10.1371/journal.pbio.0030323

**Published:** 2005-09-06

**Authors:** Gerardo A. De Blas, Carlos M Roggero, Claudia N Tomes, Luis S Mayorga

**Affiliations:** **1**Laboratorio de Biología Celular y Molecular, Instituto de Histología y Embriología, Consejo Nacional de Investigaciones Científicas y Técnicas, Facultad de Ciencias Médicas, Universidad Nacional de Cuyo, Mendoza, Argentina; Princeton UniversityUnited States of America

## Abstract

The dynamics of SNARE assembly and disassembly during membrane recognition and fusion is a central issue in intracellular trafficking and regulated secretion. Exocytosis of sperm's single vesicle—the acrosome—is a synchronized, all-or-nothing process that happens only once in the life of the cell and depends on activation of both the GTP-binding protein Rab3 and of neurotoxin-sensitive SNAREs. These characteristics make acrosomal exocytosis a unique mammalian model for the study of the different phases of the membrane fusion cascade. By using a functional assay and immunofluorescence techniques in combination with neurotoxins and a photosensitive Ca^2+^ chelator we show that, in unactivated sperm, SNAREs are locked in heterotrimeric *cis* complexes. Upon Ca^2+^ entry into the cytoplasm, Rab3 is activated and triggers NSF/α-SNAP-dependent disassembly of *cis* SNARE complexes. Monomeric SNAREs in the plasma membrane and the outer acrosomal membrane are then free to reassemble in loose *trans* complexes that are resistant to NSF/α-SNAP and differentially sensitive to cleavage by two vesicle-associated membrane protein (VAMP)–specific neurotoxins. Ca^2+^ must be released from inside the acrosome to trigger the final steps of membrane fusion that require fully assembled *trans* SNARE complexes and synaptotagmin. Our results indicate that the unidirectional and sequential disassembly and assembly of SNARE complexes drive acrosomal exocytosis.

## Abstract 2

Unidirectional and sequential disassembly and assembly of SNARE complexes drive sperm acrosomal exocytosis.

## Introduction

Regulated exocytosis is a sophisticated process that requires the specific attachment of secretory granules to the plasma membrane and the opening of fusion pores connecting the interior of the granule to the extracellular medium [1]. Several of the proteins involved have been identified and characterized by genetic approaches, reconstitution assays, and biochemical means. The current consensus paradigm for membrane fusion is based on results obtained from diverse cellular systems, ranging from yeast to neurons. For instance, the roles assigned to small GTPases of the Rab family derive from studies carried out in endocytosis models [2], whereas those of SNAREs come from neuroendocrine cell exocytosis [1]. At the core of this paradigm, Rabs promote the tethering—loose and reversible attachment—of the compartments that will fuse [3]. Subsequently, the assembly of heterotrimeric *trans* SNARE complexes brings about the docking—tight and irreversible attachment—of the fusing membranes [4]. Docking is followed by the opening and expansion of the fusion pore. In regulated exocytosis, this final stage requires an increase in cytoplasmic Ca^2+^ and the action of Ca^2+^ sensor proteins such as synaptotagmin [1]. After membrane fusion, SNAREs remain engaged in heterotrimeric *cis* complexes. Disassembly of the latter is achieved by the concerted action of α-SNAP and NSF, and is required to prepare SNAREs for subsequent rounds of fusion.

SNAREs are classified as R or Q based on the identity of a highly conserved residue [5]. Q-SNAREs and R-SNAREs contribute three and one helixes, respectively, to ternary complexes. When Q- and R-SNAREs reside on the same membrane, complexes are in a *cis,* fusion-incompetent configuration. In contrast, when Q- and R-SNAREs reside on opposite membranes, complexes are in a *trans,* fusion-competent configuration. In neurosecretory cells, exocytotic SNARE complexes are composed of syntaxin1A and a synaptosome-associated protein of 25 kD (SNAP25), which are two plasma membrane Q-SNAREs, and vesicle-associated membrane protein (VAMP) 2, which is a R-SNAREs found in secretory vesicles. These proteins are the target of botulinum and tetanus toxins, a set of highly specific zinc-dependent endoproteases [6]. In fact, the role of SNAREs in regulated exocytosis was unequivocally established thanks to the striking inhibitory effect of these neurotoxins on secretion [7]. Only when not assembled in tight complexes are SNAREs susceptible to cleavage [8], making these toxins excellent tools for the diagnosis of SNARE assembly status.

The acrosome is a large membrane-limited granule that overlies the nucleus of the mature spermatozoon [9]. Upon stimulation, sperm undergo exocytosis of this granule in a synchronized wave, with no recycling of components. Acrosomal exocytosis (AE) is an all-or-nothing event that comprises the opening of hundreds of fusion pores between the outer acrosomal membrane and the plasmalemma. AE depends on Rab3A, NSF/α-SNAP, and toxin-sensitive members of SNARE families [10–13]. It also requires an efflux of Ca^2+^ from inside the acrosome even in the presence of high cytosolic concentrations [14]. Concurrence of Rab- and toxin-sensitive, SNARE-dependent pathways is a hallmark of AE that makes it a unique mammalian model to study the different phases of the membrane fusion cascade. This feature is not found in other systems. Most exocytotic models either do not have a well defined role for, or are negatively regulated by, Rabs [1]. Likewise, systems in which Rabs are necessary for fusion typically contain toxin-insensitive SNARE isoforms.

By using a combination of neurotoxins and a photosensitive Ca^2+^ chelator, we show here that AE proceeds through a sequential set of events initiated when Rab3 is activated and triggers NSF/α-SNAP-dependent disassembly of *cis* SNARE complexes. SNAREs then reassociate in loose *trans* complexes until an efflux of intra-acrosomal Ca^2+^ promotes synaptotagmin- and SNARE-dependent membrane fusion.

## Results

### Rab3A Is Required before, and SNAREs and Synaptotagmin VI after, Intra-Acrosomal Ca^2+^ Efflux

**Figure 1:**
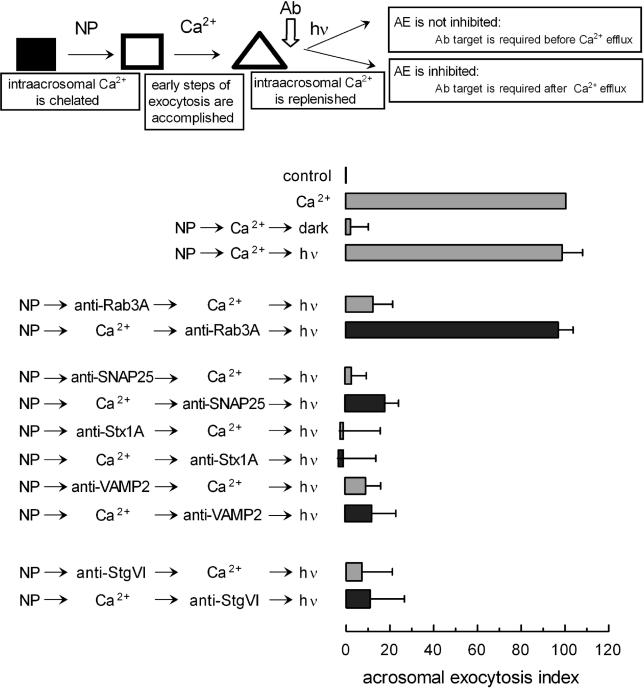
Rab3A Is Required before, and SNAREs and Synaptotagmin VI after, Intra-Acrosomal Ca^2+^ Efflux Permeabilized spermatozoa were loaded with 10 μM NP-EGTA-AM (NP) for 15 min at 37 °C to chelate intra-acrosomal Ca^2+^. AE was then initiated by adding 0.5 mM CaCl_2_ (10 μM free Ca^2+^)(Ca^2+^). After further 15 min incubation at 37 °C to allow exocytosis to proceed up to the intra-acrosomal Ca^2+^-sensitive step, sperm were treated for 15 min at 37 °C with antibodies that recognize Rab3A (20 μg/ml, anti-Rab3A), SNAP25 (20 μg/ml, anti-SNAP25), syntaxin1A (1/25 dilution, anti-Stx1A), VAMP2 (20 μg/ml, anti-VAMP2), or synaptotagmin VI (30 μg/ml, anti-StgVI). All these procedures were carried out in the dark. UV flash photolysis of the chelator was induced at the end of the incubation period (hν), and the samples were incubated for 5 min to promote exocytosis (NP→Ca^2+^→antibody→hν, black bars; a diagram of the experiment is shown at the top of the figure). Sperm were then fixed and AE was measured as described in Materials and Methods. Several controls were included (grey bars): background AE in the absence of any stimulation (control); AE stimulated by 10 μM free Ca^2+^ (Ca^2+^), inhibitory effect of NP-EGTA-AM in the dark (NP→Ca^2+^→dark), and the recovery upon illumination (NP→Ca^2+^→hν); and inhibitory effect of the antibodies when present throughout the experiment (NP→antibody→Ca^2+^→hν). The data were normalized as described in Materials and Methods (mean ± SEM). Statistical analysis is provided in Table S2.

*O*-nitrophenyl EGTA–acetoxymethyl ester (NP-EGTA-AM ) is a photolabile Ca^2+^ chelator that prevents inducer-triggered AE in sperm permeabilized by streptolysin-O (SLO); NP-EGTA-AM does this by entering the cytosol, diffusing through the outer acrosomal membrane, and accumulating inside the acrosome [14]. UV photolysis of NP-EGTA-AM rapidly replenishes the acrosomal Ca^2+^ pool, resuming exocytosis (Figure 1). In combination with AE inhibitors, NP-EGTA-AM helps to determine whether fusion-related factors are required before or after the intra-acrosomal Ca^2+^-sensitive step. Briefly, NP-EGTA-AM allows an AE inducer to prepare the fusion machinery up to the point when intra-acrosomal Ca^2+^ is required. Inhibitors are then added and the tubes illuminated. Resistance to inhibitors—reflected in unaffected exocytosis—implies that the targets of the fusion-related factors are required upstream of intra-acrosomal Ca^2+^ efflux. Sensitivity to inhibitors—revealed by blocked exocytosis—means their targets are located after the intra-acrosomal Ca^2+^-sensitive step (see Figure 1). AE is always prevented when the inhibitors are added prior to the inducer and maintained throughout the experiment. An anti-Rab3A antibody inhibited exocytosis when added before challenging with Ca^2+^ but not afterward. In contrast, antibodies against syntaxin1A, SNAP25, VAMP2, and synaptotagmin VI were able to abrogate exocytosis even when added after the inducer (Figure 1). These results indicate that Rab3A is necessary early in the fusion cascade, before Ca^2+^ is released from the acrosome, whereas SNAREs and synaptotagmin VI are required later, during or after the intra-acrosomal Ca^2+^ efflux. Similar experiments support an early role for NSF/α-SNAP [13]. These observations are summarized in Table 1.

**Table 1:**
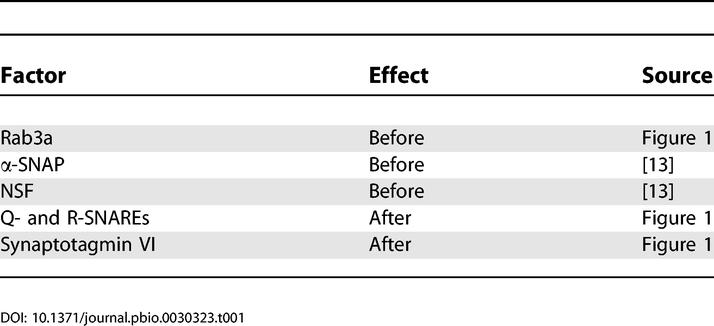
Factors Acting on Exocytosis before or after the Release of Ca^2+^ from the Acrosome

### SNAREs Are Assembled in Neurotoxin-Resistant Complexes in Resting Spermatozoa

NSF and α-SNAP are proteins that catalyze the priming of the fusion machinery, disrupting the *cis* SNARE complex and activating individual SNAREs, and are required for AE [13]. This suggests that sperm SNAREs are initially assembled in inactive *cis* complexes. Such complexes are resistant to cleavage by neurotoxins [8].

**Figure 2:**
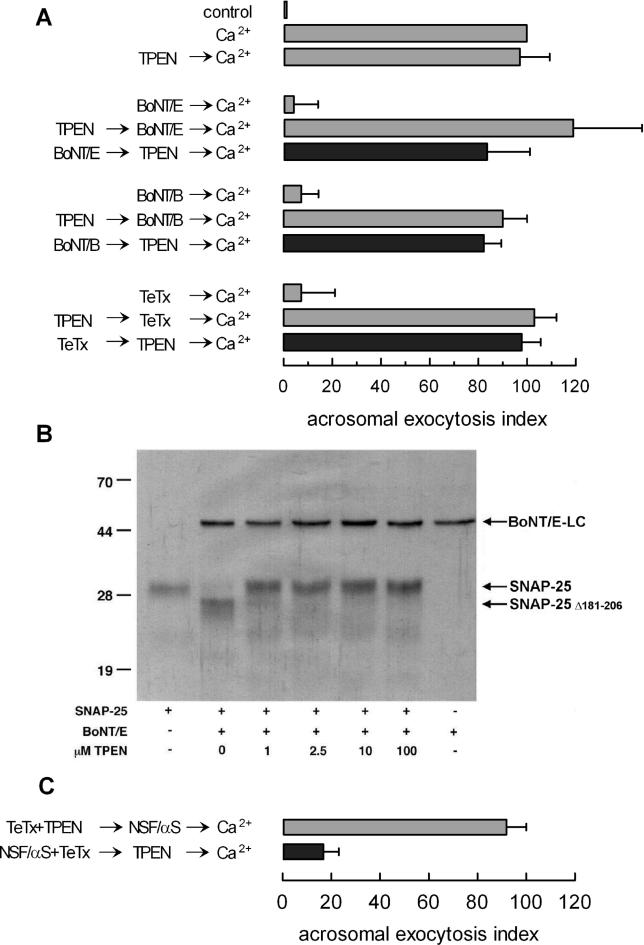
SNAREs Are Assembled in Neurotoxin-Resistant Complexes in Resting Spermatozoa (A) Permeabilized spermatozoa were treated at 37 °C for 15 min with 357 nM BoNT/E, 100 nM BoNT/B, or 100 nM TeTx. Next, 2.5 μM TPEN (see [B]) was added and AE was activated by adding 0.5 mM CaCl_2_ (10 μM free Ca^2+^) and the incubation continued for an additional 15 min (black bars). Sperm were then fixed and AE was measured as described in Materials and Methods. Several controls were included (grey bars): background AE in the absence of any stimulation (control); AE stimulated by 10 μM free Ca^2+^ (Ca^2+^); TPEN effect on exocytosis (TPEN→Ca^2+^); inhibitory effect of the neurotoxins on exocytosis (neurotoxin→Ca^2+^); and block of neurotoxin activity by TPEN (TPEN→neurotoxin→Ca^2+^). (B) Recombinant SNAP25 (0.7 μg) was incubated for 15 min at 37 °C in the presence of 0.6 μg of BoNT/E and increasing concentrations of TPEN. Samples were then resolved by SDS-PAGE and stained with Coomassie blue. Molecular weight standards are indicated on the left (in kilodaltons). Densitometry and quantitation of the stained bands show 100%, 7%, 92%, 98%, 100%, and 100% of intact SNAP25 in lanes 1–6 (from left to right), respectively. (C) Treatment with TeTx was performed as described in (A), in the presence of 310 nM NSF and 500 nM α-SNAP (NSF/αS) to promote SNARE complex dissociation (black bar). Incubation with NSF/α-SNAP in the presence of TPEN-inactivated toxin did not affect exocytosis (grey bar). The data in (A) and (C) were normalized as described in Materials and Methods (mean ± SEM). Statistical analysis is provided in Table S3.

To assess whether SNARE proteins are accessible to neurotoxins in unstimulated spermatozoa, permeabilized sperm were incubated with the light chains of botulinum neurotoxin (BoNT) E or B—BoNT/E cleaves SNAP25 and BoNT/B cleaves VAMP—or tetanus toxin (TeTx), which cleaves VAMP. Toxins were then inactivated by the specific zinc chelator N,N,N′,N′-tetrakis (2-pyridymethyl) ethylenediamine (TPEN) [15]. AE was subsequently stimulated with Ca^2+^ and secretion assessed. Exocytosis was not abrogated under these conditions (Figure 2A), suggesting that SNAP25 and VAMP2 are in a toxin-resistant configuration in resting spermatozoa.

To rule out the possibility that permeabilization would be artifactually responsible for *cis* SNARE complex formation in human sperm, we conducted experiments similar to those depicted in Figure 2A, but in intact human sperm. Because sperm were not permeabilized, holotoxins were used instead of the isolated light chains. These clostridial neurotoxins consist of a heavy subunit, responsible for binding to plasma membrane receptors (in particular gangliosides G_T1b_ and G_Q1b_), and a light subunit that carries the proteolytic activity [6]. Heavy-chain-mediated binding is required for internalization of the light chain, which acts in the cytosol [6]. Because both G_T1b_ and G_Q1b_ gangliosides have been isolated from sperm preparations [16] we expected non-permeabilized sperm to be at least partially sensitive to holotoxins. Indeed, when intact (i.e., non-permeabilized) human sperm were exposed to BoNT/A (SNAP25-specific) and BoNT/F (VAMP-specific) before triggering exocytosis with the Ca^2+^ ionophore A23187, an approximately 50% inhibition in AE was observed (Figure S1). Toxin concentrations were 5-fold higher than those required to inhibit exocytosis in permeabilized sperm [12] to allow for the poor internalization of the catalytic subunits. Once again, we found that the SNARE proteins were not accessible to neurotoxins in unstimulated cells, since exocytosis was not attenuated when toxins were inactivated by TPEN before challenging with A23187 (Figure S1), indicating that SNAP25 and VAMP were in a toxin-resistant configuration prior to initiating AE.

To show directly that TPEN blocks the proteolytic activity of neurotoxins, recombinant SNAP25 was incubated with BoNT/E in the absence or presence of increasing concentrations of the zinc chelator. As shown in Figure 2B, BoNT/E cleaved SNAP25. TPEN completely inhibited its activity at concentrations of 1 μM and higher, in total agreement with the functional data.

These results show that, in the absence of sperm stimulation, Q- and R-SNAREs are protected from neurotoxin cleavage, not adopting a toxin-sensitive state during the 15-min incubation protocol. These results were similar in permeabilized and intact sperm. Thus, we conclude that SNAREs are engaged in a toxin-resistant configuration in resting sperm. An attractive possibility is that they are locked in ternary *cis* complexes rather than undergoing cyclic assembly and disassembly like SNAREs in other systems. If this is the case, addition of an excess of recombinant NSF/α-SNAP should force disassembly, rendering the SNAREs toxin-sensitive. We therefore incubated sperm with NSF/α-SNAP in the presence of TeTx. The toxin was subsequently inactivated by addition of TPEN, and AE was stimulated. Ca^2+^ failed to elicit exocytosis, indicating that NSF/α-SNAP rendered VAMP sensitive to toxin cleavage (Figure 2C). In conclusion, our data indicate that, in resting sperm, SNAREs are in toxin-resistant *cis* complexes that can be disassembled by NSF/α-SNAP.

### Rab3A Triggers the Disassembly of SNARE Complexes before Intra-Acrosomal Ca^2+^ Efflux

**Figure 3:**
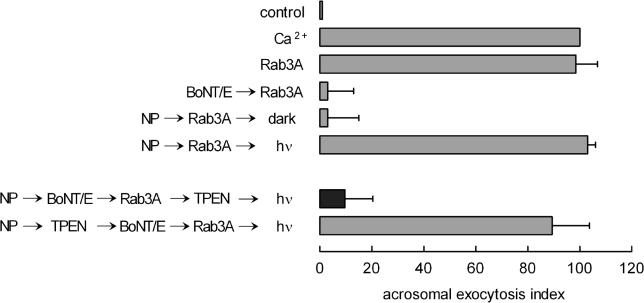
Activation with Rab3A Triggers the Disassembly of SNARE Complexes before Intra-Acrosomal Ca^2+^ Efflux Permeabilized spermatozoa were loaded with 10 μM NP-EGTA-AM (NP) for 15 min at 37 °C to chelate intra-acrosomal Ca^2+^. Then 357 nM BoNT/E was added to the system, and AE was initiated by adding 300 nM GTP-γ-S-loaded Rab3A. After 15 min at 37 °C, the toxin was inactivated with 2.5 μM TPEN, and photolysis of NP was induced by UV illumination (hν). The samples were incubated for a further 5 min to promote exocytosis (NP→BoNT/E→Rab3A→TPEN→hν, black bar). At the end of the incubation, sperm were fixed and AE was measured as described in Materials and Methods. Several controls are included (grey bars): background AE in the absence of any stimulation (control); AE stimulated by 10 μM free Ca^2+^ (Ca^2+^) or Rab3A (Rab3A); blockage of Rab3A-triggered exocytosis by BoNT/E (BoNT/E→Rab3A); inhibitory effect of NP-EGTA-AM in the dark (NP→Rab3A→dark) and the recovery upon illumination (NP→Rab3A→hν); and inactivation of the neurotoxin by TPEN (NP→TPEN→BoNT/E→Rab3A→hν). The data were normalized as described in Materials and Methods (mean ± SEM). Statistical analysis is provided in Table S4.

In our permeabilized sperm model, exocytosis is achieved when an increase in the cytosolic Ca^2+^ concentration promotes the activation of Rab3A [10]. Alternatively, AE can be initiated by adding recombinant Rab3A preloaded with GTP-γ-S [11]. Rab3A-triggered exocytosis is sensitive to neurotoxins [12]. Therefore, at some point after Rab3A activation, SNAREs must pass from a toxin-resistant to a toxin-sensitive state. This transition may occur before or after the release of Ca^2+^ from the acrosome. To distinguish between these two possibilities, intra-acrosomal Ca^2+^ was chelated with NP-EGTA-AM and AE was initiated with Rab3A in the presence of BoNT/E. The toxin was then inactivated by TPEN and intra-acrosomal Ca^2+^ replenished by NP-EGTA-AM photolysis. As shown in Figure 3, exocytosis was efficiently inhibited, indicating that SNAREs were disassembled—because SNAP25 was cleaved—after Rab3A activation and before the intra-acrosomal Ca^2+^ efflux. In brief, Rab3A—and not the efflux of intra-acrosomal Ca^2+^—elicited SNARE complex disassembly. It is worth noting that disassembly appears to be Ca^2+^-independent, since it occurred at a low Ca^2+^ concentration in the medium (approximately 100 nM [14]).

### Immunofluorescence Experiments Indicate That SNAREs Are Disassembled by Sperm Activation

**Figure 4:**
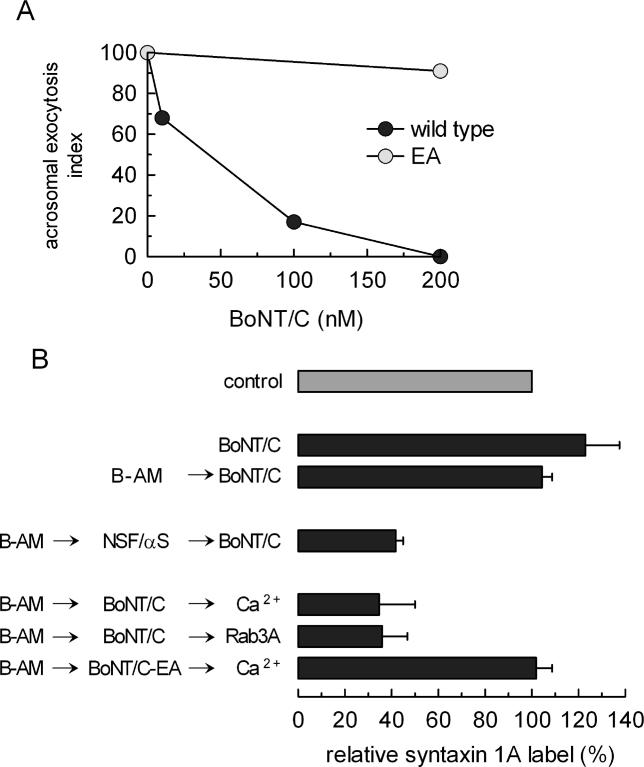
Syntaxin1A Is Assembled in Toxin-Resistant Complexes That Are Disassembled by NSF/α-SNAP or by Sperm Activation (A) Permeabilized spermatozoa were incubated for 15 min at 37 °C with increasing concentrations of BoNT/C (black circles, wild type; grey circles, EA, a protease-inactive mutant) and then stimulated with 10 μM Ca^2+^ for 15 min at 37 °C. Afterwards, sperm were fixed and AE measured as described in Materials and Methods. (B) To assess the assembly state of syntaxin1A, sperm were incubated with 100 nM BoNT/C (15 min at 37 °C), and the cells were then fixed and immunostained with an anti-syntaxin1A antibody recognizing an epitope that is cleaved by the toxin. To prevent AE, which would release syntaxin into the medium by vesiculation of the acrosome, intra-acrosomal Ca^2+^ was chelated with 10 μM BAPTA-AM (15 min at 37 °C, B-AM). The toxin treatment in resting sperm (BoNT/C) or B-AM-loaded sperm (B-AM→BoNT/C) had no effect on the syntaxin labeling compared to untreated sperm (control). However, when 310 nM NSF and 500 nM α-SNAP were added to the system to promote the disassembly of SNARE complexes, the toxin significantly decreased the syntaxin labeling (B-AM→NSF/αS→BoNT/C). The BoNT/C treatment also affected syntaxin labeling when sperm were stimulated for 15 min at 37 °C with 10 μM free Ca^2+^ (B-AM→ BoNT/C→Ca^2+^) or 300 nM Rab3A (B-AM→BoNT/C→Rab3A). The protease-inactive mutant did not affect labeling under these conditions (B-AM→BoNT/C-EA→Ca^2+^). Fluorescence was normalized as described in Materials and Methods. The data represent the mean ± SEM. Statistical analysis is provided in Table S5.

Neurotoxins are proteases; hence, their effect on sperm SNAREs should be reflected by immunostaining, provided that anti-SNARE antibodies recognizing the removed portions are used as probes. BoNT/C was selected for these experiments for the following reasons: (i) it cuts syntaxin1A near the transmembrane domain, eliminating most of the cytoplasmic region [6]; (ii) a mutant without catalytic activity is available (BoNT/C–E230A [EA], T. Binz, personal communication); and (iii) immunolabeling with a polyclonal antibody that recognizes the region of the protein cleaved by the toxin is highly reproducible. When BoNT/C was introduced into our assay, substantial inhibition of exocytosis was observed at low concentrations of the toxin (Figure 4). As expected, the protease-null mutant did not affect AE.

**Figure 5:**
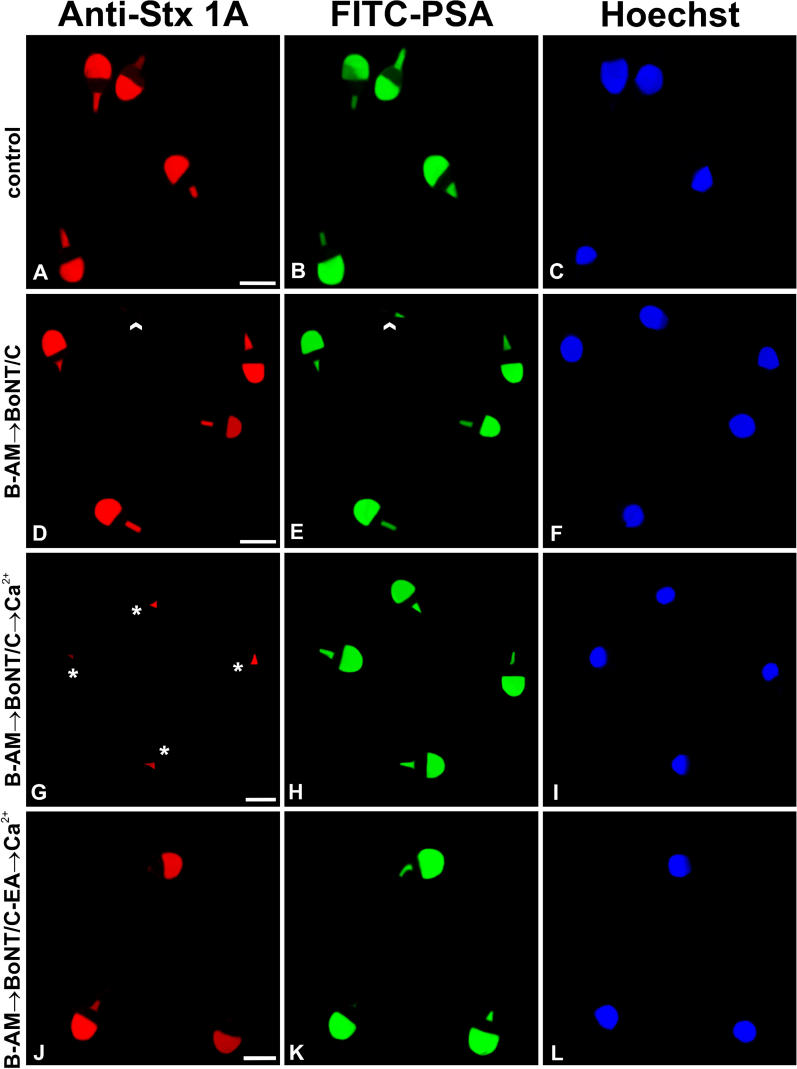
Effect of BoNT/C on Syntaxin1A Immunofluorescence Sperm were incubated with 100 nM BoNT/C (15 min at 37 °C) as explained in Figure 4. The cells were then fixed and triple-stained with an anti-syntaxin1A antibody that recognizes an epitope trimmed by the toxin (red; [A, D, G, and J]), FITC-PSA to differentiate between reacted and intact sperm (green; [B, E, H, and K]), and Hoechst 33258 to visualize all cells in the field (blue; [C, F, I, and L]). Notice that spontaneously reacted sperm were negative for syntaxin1A staining (arrowheads in [D] and [E]). BoNT/C had no effect on resting sperm (compare [A–C] with [D–F]). However, labeling in sperm stimulated with 10 μM Ca^2+^ in the presence of BAPTA-AM to prevent exocytosis (observe that PSA staining is not affected) was significantly reduced by the toxin (asterisks, [G]). In contrast, the same experimental condition in the presence of the protease-inactive toxin (BoNT/C-EA) had no effect (J–L). Bars = 5 μm.

For immunofluorescence experiments, sperm were incubated under different conditions with or without BoNT/C. Cells were fixed and double-labeled with fluorescein isothiocyanate (FITC)–*Pisum sativum* agglutinin (PSA)—to distinguish between reacted and intact spermatozoa—and with an anti-syntaxin1A antibody (Figure 5). Under control conditions (incubation without BoNT/C), clear immunolabeling was observed in the acrosomal region of most cells (Figure 5A). This pattern is expected for a protein that participates in sperm exocytosis. Notice that spontaneously reacted sperm (not stained by FITC-PSA) did not exhibit syntaxin staining (Figure 5D and 5E). Addition of BoNT/C did not decrease the immunofluorescence labeling with the anti-syntaxin1A antibody (Figures 4B and 5D), indicating that this SNARE was protected from toxin cleavage in resting spermatozoa. Addition of NSF/α-SNAP in the presence of the toxin caused a significant decrease in the percentage of syntaxin-labeled cells (Figure 4B). Because these proteins disassemble SNARE complexes, their ability to confer BoNT/C vulnerability—marked by diminished immunolabeling—means that syntaxin is engaged in resistant complexes in untreated sperm. To verify that sperm activation triggers SNARE disassembly in a step previous to the Ca^2+^ efflux from the intra-acrosomal pool, this store was depleted with BAPTA-AM, and sperm exocytosis stimulated with Ca^2+^ or Rab3A in the presence of BoNT/C. Under these conditions, the percentage of sperm with acrosomal syntaxin1A labeling decreased significantly, indicating that the protein became toxin-sensitive after sperm activation and before the efflux of intra-acrosomal Ca^2+^ (Figures 4B and 5G). In contrast, stimulation in the presence of catalytically inactive BoNT/C caused no decrease in the percentage of labeled spermatozoa (Figures 4B and 5J), ruling out the possibility of an effect unrelated to the proteolytic activity of the toxin. FITC-PSA staining was not affected by the different conditions, indicating that the decrease in syntaxin1A immunolabeling was not due to AE that would have released the outer acrosomal membrane together with the apposed plasma membrane (Figure 5).

These results reaffirm the notion that SNAREs are toxin-protected in resting spermatozoa and that addition of NSF/α-SNAP unprotects them. Furthermore, activation of spermatozoa (with either Ca^2+^ or Rab3A) under conditions that deplete intra-acrosomal Ca^2+^ promotes their cleavage by BoNTs, indicating that *cis* SNARE complexes are disassembled at a step prior to the intra-acrosomal Ca^2+^ efflux.

### SNAREs Are Not Engaged in Tight Complexes before the Efflux of Intra-Acrosomal Ca^2+^

**Figure 6:**
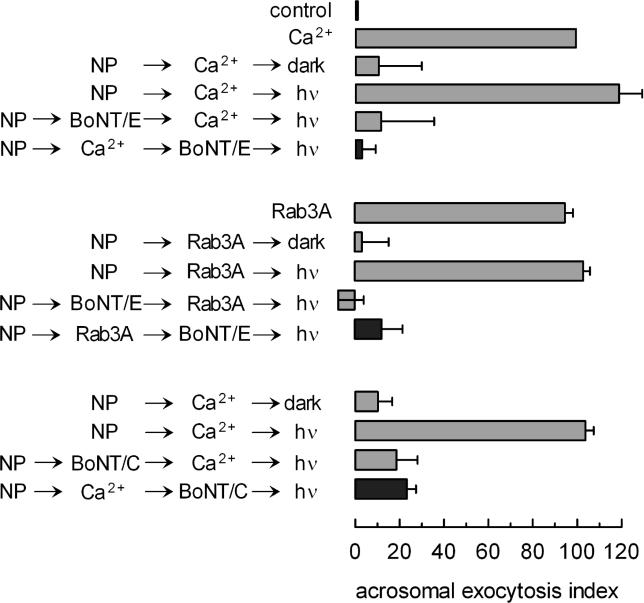
SNAREs Do Not Reassemble in Tight Complexes before the Efflux of Intra-Acrosomal Ca^2+^ Permeabilized spermatozoa were loaded with 10 μM NP-EGTA-AM (NP) for 15 min at 37 °C to chelate intra-acrosomal Ca^2+^. AE was then initiated by adding 10 μM free Ca^2+^ (Ca^2+^) or 300 nM Rab3A (Rab3A). After 15 min incubation at 37 °C to allow exocytosis to proceed to the intra-acrosomal Ca^2+^-sensitive step, neurotoxins recognizing SNAP25 (BoNT/E) and syntaxin (BoNT/C) were added to the tubes to assess whether the SNAREs had reassembled into toxin-resistant complexes. Intra-acrosomal Ca^2+^ was replenished by photolysis of NP (hν), and samples were incubated for 5 min to promote exocytosis (NP→Ca^2+^/Rab3A→neurotoxins→hν, black bars). Sperm were then fixed and AE measured as described in Materials and Methods. Several controls are included (grey bars): background AE in the absence of any stimulation (control); AE stimulated by 10 μM free Ca^2+^ (Ca^2+^) or Rab3A (Rab3A); inhibitory effect of NP-EGTA-AM in the dark (NP→Ca^2+^/Rab3A→dark) and recovery upon illumination (NP→Ca^2+^/Rab3A→hν); and AE inhibition when neurotoxins were present throughout the incubation (NP→neurotoxins→Ca^2+^/Rab3A→hν). The data were normalized as described in Materials and Methods (mean ± SEM). Statistical analysis is provided in Table S6.

Upon sperm stimulation, *cis* SNARE complexes disassemble and pass through a toxin-sensitive configuration. The next question we answered was whether they reassemble into toxin-resistant complexes before Ca^2+^ efflux from the acrosome triggers the final steps of exocytosis. For these experiments, sperm were stimulated with Ca^2+^ or Rab3A in the presence of NP-EGTA-AM. BoNT/E (or BoNT/C) was then added to the assay to cleave unprotected SNAP25 (or syntaxin). Finally, intra-acrosomal Ca^2+^ was replenished by photolysis of the chelator. BoNT/E inhibited both Ca^2+^- and Rab3A-triggered exocytosis even when added after AE had progressed to the intra-acrosomal Ca^2+^-sensitive step (Figure 6). A similar result was observed with BoNT/C (Figure 6). We conclude that SNAREs were not protected before the efflux of intra-acrosomal Ca^2+^; hence, they may remain as monomers after they are disassembled following sperm activation. Alternatively, they may reassemble partially in loose *trans* complexes that also are sensitive to neurotoxins. These complexes have been postulated in secretory vesicles and granules already attached to the plasma membrane. In the loose complexes Q- and R-SNAREs are contributed by different membranes but are not tightly packed. Hence, they can be cleaved by most neurotoxins, including BoNT/E and BoNT/C [17,18].

### SNAREs Are Forming Loose *trans* Complexes before the Efflux of Intra-Acrosomal Ca^2+^

**Figure 7:**
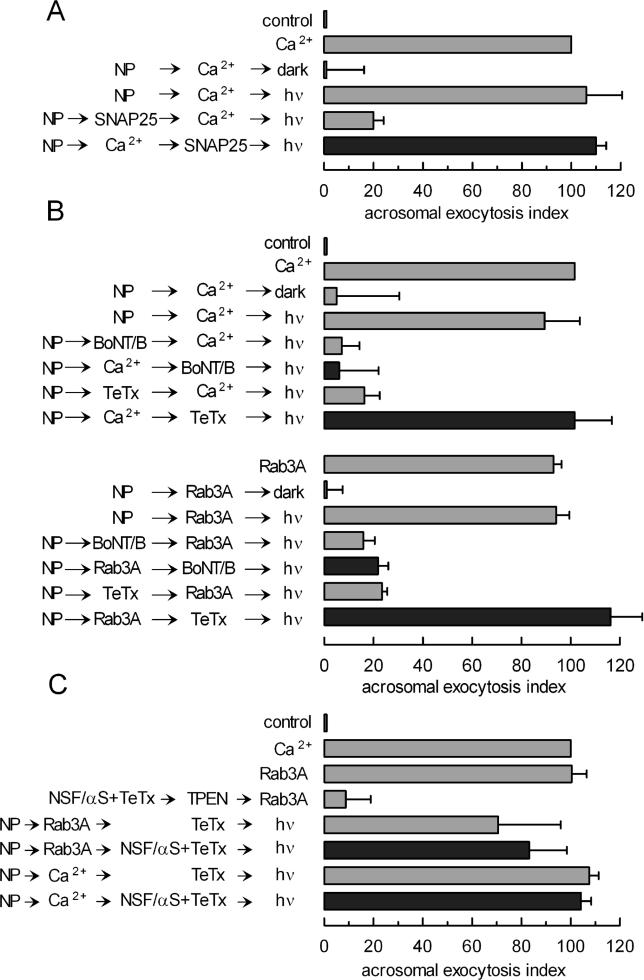
SNAREs Reassemble in Loose Complexes That Are Resistant to NSF/α-SNAP before the Efflux of Intra-Acrosomal Ca^2+^ (A) Permeabilized spermatozoa were loaded with 10 μM NP-EGTA-AM (NP) for 15 min at 37 °C to chelate intra-acrosomal Ca^2+^. AE was then initiated by adding 10 μM free Ca^2+^ (Ca^2+^). After 15 min incubation at 37 °C to allow exocytosis to proceed to the intra-acrosomal Ca^2+^-sensitive step, 800 nM recombinant SNAP25 (SNAP25) was added to compete with endogenous SNAP25. Intra-acrosomal Ca^2+^ was replenished by photolysis of NP-EGTA-AM (hν), and the samples were incubated for 5 min to promote exocytosis (NP→Ca^2+^→SNAP25→hν, black bar). Sperm were then fixed and AE was measured as described in Materials and Methods. (B) Permeabilized spermatozoa were loaded with 10 μM NP-EGTA-AM (NP) for 15 min at 37 °C. AE was then initiated by adding 10 μM free Ca^2+^ (Ca^2+^) or 300 nM Rab3A (Rab3A). After 15 min incubation at 37 °C, 100 nM neurotoxin recognizing VAMP (BoNT/B and TeTx) was added to the tubes to assess whether the SNAREs had reassembled in loose *trans* complexes sensitive to BoNT/B but not to TeTx. After 15 min incubation at 37 °C, intra-acrosomal Ca^2+^ was replenished by photolysis of NP-EGTA-AM (hν), and the samples were incubated for 5 min to promote exocytosis (NP→Ca^2+^/Rab3A→neurotoxin→hν, black bars). Sperm were then fixed and AE measured as described in Materials and Methods. (C) To assess whether NSF/α-SNAP can disassemble loose *trans* SNARE complexes, permeabilized sperm treated as in (B) were incubated with TeTx in the presence of 310 nM NSF and 500 nM α-SNAP (NP→Ca^2+^/Rab3A→NSF/αS+TeTx→hν, black bars). Several controls were included in (A), (B), and (C) (grey bars): background AE in the absence of any stimulation (control); AE stimulated by 10 μM free Ca^2+^ (Ca^2+^) or 300 nM Rab3A (Rab3A); inhibitory effect of NP-EGTA-AM in the dark (NP→Ca^2+^/Rab3A→dark) and the recovery upon illumination (NP→Ca^2+^/Rab3A→hν); inhibitory effect when SNAP25 was present throughout the incubations (NP→SNAP25→Ca^2+^→hν); inhibitory effect when the neurotoxins were present throughout the incubations (NP→neurotoxin→Ca^2+^/Rab3A→hν); and the effect of NSF/α-SNAP on SNARE complexes in unstimulated sperm (NSF/αS+TeTx→TPEN→Rab3A→hν). The data were normalized as described in Materials and Methods (mean ± SEM). Statistical analysis is provided in Table S7.

Following *cis* complex disassembly during sperm activation, SNAREs remain toxin-sensitive until Ca^2+^ efflux from the acrosome triggers the last steps of AE. Both monomeric and heterotrimeric loose *trans* configurations are susceptible to toxin cleavage. To distinguish between the two, we used recombinant soluble (unpalmitoylated) SNAP25. This protein efficiently inhibits AE, most likely because it competes with endogenous, membrane-associated SNAP25, for the formation of productive SNARE complexes [12]. We reasoned that recombinant SNAP25 would be able to compete with the endogenous protein when monomeric, but not when engaged in complexes. Sperm were stimulated with Ca^2+^ in the presence of NP-EGTA-AM, recombinant SNAP25 was added, and intra-acrosomal Ca^2+^ replenished by UV photolysis. Under these conditions, recombinant SNAP25 was not able to inhibit exocytosis (Figure 7A). These observations suggest that after stimulation and before the Ca^2+^ efflux from the acrosome, endogenous SNAREs are engaged in SNARE complexes.

Differential sensitivity to BoNT/B and TeTx constitutes an independent approach to distinguish between monomeric VAMP and that engaged in loose *trans* complexes. These toxins cleave the same peptide bond, exposed in both configurations [6]. Interestingly, TeTx binds to the N-terminus of the VAMP coil domain whereas BoNT/B binds the C-terminus. Since SNARE complex assembly begins at the N-terminus, the TeTx-recognition site is hidden in loose SNARE complexes while the BoNT/B recognition site is exposed. In other words, TeTx can only cleave monomeric VAMP while BoNT/B also cuts VAMP loosely assembled in SNARE complexes [19]. As shown in Figure 7B, BoNT/B—but not TeTx—was capable of inhibiting AE when the system was stimulated with Ca^2+^ and allowed to reach the intra-acrosomal Ca^2+^-sensitive step. To assess whether loose *trans* complex assembly takes place in the absence of added Ca^2+^, Rab3A was used as an inducer in the presence of EGTA. Again, both toxins blocked exocytosis when present from the beginning of the experiment, but only BoNT/B inhibited when added after stimulation (Figure 7B). These data suggest that assembly of *trans* SNARE complexes is Ca^2+^-independent.

The fact that exocytosis was accomplished in the constant presence of exogenous SNAP25 and TeTx (Figure 7A and 7B) indicates that *trans* SNARE complexes are not disassembled after intra-acrosomal Ca^2+^ efflux. We hypothesized that after sperm stimulation, SNAREs remain irreversibly engaged in loose *trans* complexes as long as intra-acrosomal Ca^2+^ is kept unavailable. As shown in Figure 2C, recombinant NSF/α-SNAP can disassemble *cis* SNARE complexes in unstimulated spermatozoa. We asked whether they could also disengage loose *trans* complexes. To this end, NP-EGTA-AM-loaded sperm were stimulated with Ca^2+^ or Rab3A and then treated with TeTx in the presence or absence of NSF/α-SNAP. The results, presented in Figure 7C, show that these chaperones failed to confer TeTx sensitivity when added after the exocytic process had progressed to the intra-acrosomal Ca^2+^-sensitive step. These observations indicate that *trans* SNARE complexes cannot be disassembled by NSF/α-SNAP. The notion that *trans* SNARE complexes are resistant to NSF/α-SNAP opposes the original SNARE hypothesis [20] and, although suggested in the past [21], has to our knowledge never before been shown in actual cells.

### Immunofluorescence Experiments Show That VAMP2 Is Engaged in Loose *trans* SNARE Complexes after Stimulation

**Figure 8:**
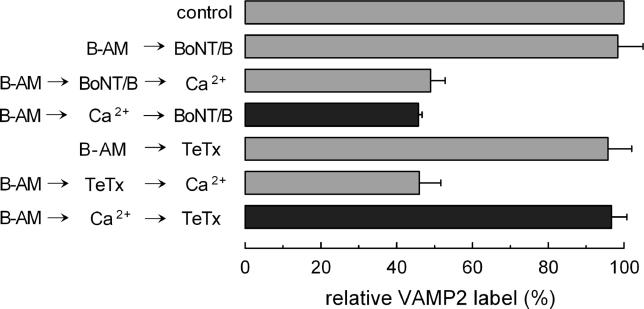
VAMP2 Is Engaged in Loose SNARE Complexes before the Efflux of Intra-Acrosomal Ca^2+^ Permeabilized spermatozoa were loaded with 10 μM BAPTA-AM (B-AM) for 15 min at 37 °C to chelate intra-acrosomal Ca^2+^. AE was then initiated by adding 10 μM free Ca^2+^ (Ca^2+^). After 15 min incubation at 37 °C to allow exocytosis to proceed to the intra-acrosomal Ca^2+^-sensitive step, 100 nM neurotoxins recognizing VAMP (BoNT/B or TeTx) were added to the tubes and the samples were incubated for 15 min at 37 °C (B-AM→Ca^2+^→neurotoxin, black bars). Samples were then immunolabeled with an anti-VAMP2 antibody as described in Materials and Methods. Notice that at this stage VAMP2 immunolabeling was sensitive to BoNT/B but not to TeTx. Several other conditions are included (grey bars). The toxins did not affect VAMP2 staining in resting sperm (compare control versus B-AM→neurotoxin). However, the toxins decreased the VAMP2 labeling when present during stimulation (B-AM→neurotoxin→Ca^2+^). Fluorescence was normalized as described in Materials and Methods (mean ± SEM). Statistical analysis is provided in Table S8.

**Figure 9:**
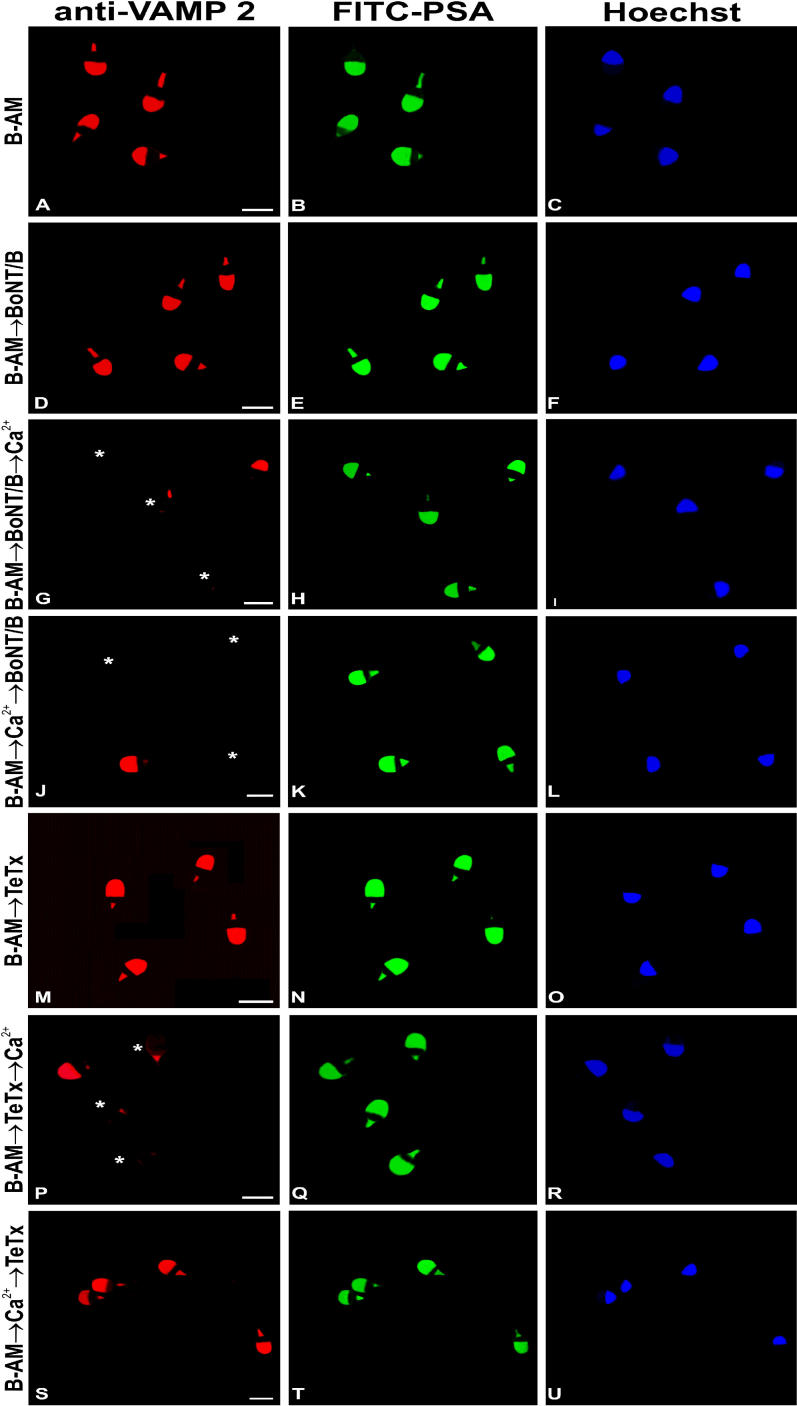
Effect of BoNT/B and TeTx on VAMP2 Immunofluorescence Sperm were incubated with 100 nM BoNT/B or TeTx (15 min at 37 °C) as described in Figure 8. The cells were then triple-stained with an anti-VAMP2 antibody that recognizes an epitope that is cleaved by the toxin (red; [A, D, G, J, M, P, and S]), FITC-PSA to differentiate between reacted and intact sperm (green; [B, E, H, K, N, Q, and T]), and Hoechst 33258 to visualize all cells in the field (blue; [C, F, I, L, O, R, and U]). BoNT/B and TeTx had no effect on resting sperm (compare [D–F] and [M–O] with [A–C]). However, labeling in sperm stimulated with 10 μM Ca^2+^ in the presence of BAPTA-AM to prevent exocytosis (observe that the PSA staining is not affected) was significantly reduced by the toxins (asterisks, [G] and [P]). In contrast, when cells were first allowed to arrive at the intra-acrosomal Ca^2+^-sensitive step and then treated with toxins, BoNT/B caused a significant decrease of the VAMP2 label (asterisks, [J]), whereas TeTx had no effect (S). Bars = 5 μm.

As shown in Figures 4B and 5, immunofluorescence is a powerful technique to monitor SNARE assembly status as reflected by SNARE sensitivity to cleavage by neurotoxins. We thus made use of the differential sensitivity of VAMP2 to TeTx and BoNT/B when engaged in loose complexes to show by VAMP2 immunostaining that these complexes form after sperm stimulation. BAPTA-AM was used in all cases to chelate intra-acrosomal Ca^2+^ and prevent VAMP2-containing membrane loss inherent to the acrosome reaction. As expected, acrosomal labeling for VAMP2 was not affected by incubating unstimulated sperm with TeTx or BoNT/B (Figures 8 and 9), consistent with the notion that VAMP2 is protected in *cis* SNARE complexes. A significant decrease in the percentage of cells exhibiting acrosomal staining was observed when toxin-loaded sperm were challenged with Ca^2+^ (Figures 8, 9G, and 9P). Thus, VAMP2 sensitization to cleavage was probably a consequence of *cis* SNARE disassembly by the stimulant. In contrast, when toxins were added after Ca^2+^ (i.e., after AE had progressed to the intra-acrosomal Ca^2+^-sensitive step), VAMP2 labeling was attenuated by BoNT/B (Figures 8 and 9J) but not by TeTx (Figures 8 and 9S). Loss of VAMP2 immunostaining following BoNT/B treatment is due to lack of immunostaining of the cleavage product (aa 1–76) by the anti-VAMP2 antibody [22]. This pattern of VAMP2 sensitivity to BoNT/B coupled to resistance to TeTx implies its engagement in loose *trans* SNARE complexes. In conclusion, immunofluorescence data further support the idea that, following sperm stimulation, SNAREs are engaged in loose *trans* complexes awaiting the release of intra-acrosomal Ca^2+^ that will trigger the final steps of AE.

## Discussion

Exocytosis of the acrosome is a synchronized, all-or-nothing process that happens only once in the life of the spermatozoon and depends on both Rab3 activation and neurotoxin-sensitive SNAREs. Because of these special features, it constitutes a particularly attractive system to examine molecular aspects of regulated exocytosis that are not amenable to experimental manipulation in other mammalian models. These include the coupling between Rab and SNARE functions, the properties of loose *trans* SNARE complexes, the role of synaptotagmin in the dynamics of SNARE assembly, and the role of intravesicular Ca^2+^ in membrane fusion. Furthermore, AE is a central process in sperm physiology, and understanding the molecular mechanisms underlying it will be of outstanding importance for our ability to regulate fertilization. Sperm contact with glycoproteins in the zona pellucida of the oocyte leads to the opening of store-operated Ca^2+^ channels in the sperm plasmalemma followed by a massive entry of Ca^2+^ from the medium into the cytoplasm, leading to exocytosis [23]. Our SLO-permeabilized sperm model, in which Ca^2+^ can freely permeate into the cells, resembles the state of open store-operated Ca^2+^ channels in intact cells and is therefore suitable to study stages of exocytosis occurring downstream of store-operated Ca^2+^ channel opening.

SNAREs are required in multiple fusion events mandatory for cell survival even under resting conditions. The ratio of monomeric to assembled SNAREs depends on the type and physiological condition of the cell. Thus, while some studies indicate that most SNAREs are free in the plasma membrane [24], others suggest that they are engaged in complexes [25,26]. SNAP25 and syntaxins can form partial all-Q complexes (three helix bundles from one SNAP25 and one syntaxin; [25]) or complexes composed of four helix bundles from two syntaxins and one SNAP25 [27]. Pre-association of Q-SNAREs in a three-bundle complex creates the docking site for the cognate R-SNARE [28]. Unlike their ternary counterparts, binary complexes are unstable and sensitive to neurotoxins [25]. Whatever the steady-state configuration of SNAREs in neuroendocrine cells might be, exocytosis is blocked by neurotoxins, suggesting that SNAREs go through toxin-sensitive stages [18,29]. In resting human sperm, both R- and Q-SNAREs are protected from toxin cleavage. Susceptibility to toxins is conferred by both endogenous or exogenously added NSF/α-SNAP. Thus, we conclude that sperm SNAREs are locked in ternary complexes in a *cis* configuration, in contrast to cells with active vesicular recycling. Because sperm have only one chance to fertilize the oocyte, tight spatial and temporal regulation of the acrosome reaction is a prerequisite for their success. In this scenario, it is not surprising that SNAREs are bound in an inactive state until exocytosis is triggered.

The connection between the rise in cytosolic Ca^2+^ occurring upon sperm stimulation and Rab3A activation is not clear. A calmodulin-mediated effect is possible, since Ca^2+^/calmodulin binds Rab3A [30] and promotes the exchange of GDP for GTP in the Rab3A–GDP dissociation inhibitor complex [31]. Although we have shown that calmodulin has an inhibitory effect on AE in permeabilized sperm and that this effect is not mediated by binding to Rab3A [32], our results do not exclude a role for calmodulin in Rab3A activation. Once Rab3A is activated, *cis* SNAREs are disassembled by NSF/α-SNAP and become accessible to neurotoxins. In other systems, active Rabs recruit a variety of effectors that tether the membranes that will fuse. NSF has been found associated with tethering complexes [33]. Moreover, NSF binds several Rabs, including Rab3A [34]. Perhaps similar interactions favor the dissociation of *cis* SNAREs by sperm NSF/α-SNAP following Rab3A activation. The proximity of the tethered membranes plus the availability of free Q- and R-SNAREs would allow their association in loose *trans* complexes. The latter can form at the very low Ca^2+^ concentrations achieved by EGTA and NP-EGTA-AM chelating Ca^2+^ in the medium/cytosol and in the acrosome, respectively. While not required for the assembly of SNARE complexes from pure proteins [27], Ca^2+^ appears to be necessary for *trans* SNARE pairing during exocytosis in PC12 cells [35]. Perhaps this incongruity is due to the presence of specific regulatory proteins in these cells. We have shown that *trans* complexes are resistant to NSF/α-SNAP, in agreement with results obtained in a proteoliposome fusion assay [21]. In contrast, *trans* SNAREs can be dissociated by the NSF/α-SNAP homologs Sec18p/Sec17p in a yeast vacuole fusion assay [36]. The reasons for this discrepancy are unknown, but it is worth mentioning that both the models (yeast vacuole fusion versus mammalian regulated exocytosis) and the experimental readout (coimmunoprecipitation of SNAREs versus toxin sensitivity) are quite different. Resistance to NSF/α-SNAP, SNAP25, and TeTx indicates that, following sperm stimulation, SNAREs are engaged in *trans* complexes that do not spontaneously revert to the monomeric configuration from which such complexes arose. Temporally, the arrest of exocytosis until Ca^2+^ is released from the acrosome correlates with SNAREs being assembled in loose *trans* complexes. Resistance to NSF/α-SNAP, SNAP25, and TeTx might also be explained if SNAREs were dispensable for the final fusion steps, as has been suggested for yeast vacuole fusion and exocytosis of cortical granules of sea urchin oocytes [36,37]. Our data do not support this view, however, since BoNTs and antibodies to SNAREs continue to prevent exocytosis downstream of intra-acrosomal Ca^2+^ release. This indicates that SNAREs are required late in the exocytotic cascade.

A direct role for intravesicular Ca^2+^ in membrane fusion has been proposed in several transport events, including the exocytosis of secretory granules [38,39]. Why would Ca^2+^ efflux from the acrosome be necessary for exocytosis? We favor the idea that tight apposition of the membranes near the forming fusion pore prevents free accessibility to cytosolic Ca^2+^. Thus, a local release from the acrosome is necessary to activate the final steps of membrane fusion. Synaptotagmins are likely involved in this late Ca^2+^-sensitive step. Synaptotagmin VI, at least, is present in the acrosomal membrane of human sperm, and this protein is required at a step downstream of the intra-acrosomal Ca^2+^ efflux (Figure 1 and [40]). Therefore, synaptotagmin VI/Ca^2+^ may favor the full zippering of SNARE complexes and thus enable membrane fusion [41].

**Figure 10:**
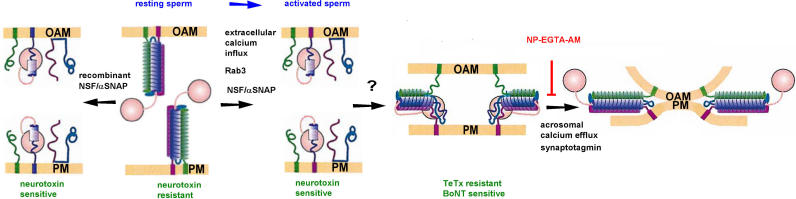
Working Model for the Dynamics of SNARE Assembly and Disassembly during AE The resistance to neurotoxin proteolysis is indicated as determined experimentally here. The block by intra-acrosomal Ca^2+^ chelators is marked in red. OAM, outer acrosomal membrane; PM, plasma membrane. See text for more details (SNARE drawings were modified from [4]).

Our data fit the working model depicted in Figure 10, in which, for simplicity, only SNAREs are shown. Initially, SNAREs are locked in inactive *cis* complexes on plasma and outer acrosomal membranes. Rab3A is activated upon Ca^2+^ entrance into the cytoplasm, triggering the tethering of the acrosome to the plasma membrane. Next, NSF/α-SNAP disassemble *cis* SNARE complexes on both membranes. Monomeric SNAREs are free to assemble in loose *trans* complexes, causing the irreversible docking of the acrosome to the plasma membrane. At this point, Ca^2+^ is released from inside the acrosome through inositol 1,4,5-trisphosphate–sensitive Ca^2+^ channels to trigger the final steps of membrane fusion, which require SNAREs (presumably in tight *trans* complexes) and synaptotagmin.

## Materials and Methods

### Reagents

SLO was obtained from Corgenix (Peterborough, United Kingdom). Gamete preparation medium (Serono, Aubonne, Switzerland) was used to culture spermatozoa. TPEN, NP-EGTA-AM, Hoechst 33258, and BAPTA-AM were purchased from Molecular Probes (Eugene, Oregon, United States). Anti-VAMP2 (mouse monoclonal, clone 69.1, purified IgG), and anti-syntaxin1A (rabbit polyclonal, whole serum) were from Synaptic Systems (Göttingen, Germany). Anti-Rab3A (rabbit polyclonal, purified IgG) was from Santa Cruz Biotechnology (Santa Cruz, California, United States). Anti-SNAP25 (mouse monoclonal, clone SP12, purified IgG) was from Stress Gen (Victoria, British Columbia, Canada). The anti–synaptotagmin VI (rabbit polyclonal, affinity purified) has been previously described [42]. TRITC-conjugated goat anti-mouse and anti-rabbit IgG were from Kirkegaard and Perry Laboratories (Gaithersburg, Maryland, United States). All other chemicals were analytical-grade and were purchased from Sigma Chemical (St. Louis, Missouri, United States) or ICN Biochemicals (Aurora, Ohio, United States).

### Recombinant proteins

Recombinant SNAP25-His_6_ was generously provided by U. Matti (Physiologisches Institut, Universität des Saarlandes, Homburg, Germany). Plasmids encoding His_6_-α-SNAP and His_6_-NSF in pQE9 (Qiagen, Valencia, California, United States) were a kind gift from S. Whiteheart (University of Kentucky, Lexington, Kentucky, United States). Plasmids encoding the light chain of BoNT/C, BoNT/C-EA, BoNT/B, and TeTx in pQE3 (Qiagen), and BoNT/E in pQE9 were generously provided by T. Binz (Medizinische Hochschule Hannover, Hannover, Germany). DNA encoding His_6_-α-SNAP was transformed into *Escherichia coli* XL1-Blue (Stratagene, La Jolla, California, United States) and induced overnight at 20 °C with 0.2 mM IPTG. The same protocol was used for the expression of BoNT/E, BoNT/C, BoNT/C-EA, and TeTx. Plasmid constructs encoding NSF and BoNT/B were transformed into *E. coli* M15pRep4 (Qiagen) and induced 4 h at 30 °C with 1 mM IPTG. Purification of recombinant proteins was accomplished according to [43], except that 0.5 mM ATP, 5 mM MgCl_2_, and 2 mM DTT were added to all buffers involved in the purification of His_6_-NSF. The expression plasmid pGEX2T containing the cDNA-encoding human Rab3A was generously provided by M. Colombo and P. Stahl (Washington University, St. Louis, Missouri, United States). GST-Rab3A was expressed in *E. coli* XL1-Blue, purified, prenylated, and loaded with guanosine 5′-O-3-thiotriphosphate following standard procedures [11].

### AE in permeabilized sperm

Human semen samples were obtained from normal healthy donors, after at least 2 d of abstinence. Highly motile sperm were recovered following a swim-up separation for 1 h in gamete preparation medium at 37 °C in an atmosphere of 5% CO_2_/95% air. Concentration was adjusted to 5–10 × 10^6^/ml, and incubation proceeded for at least 2 h under conditions that supported capacitation (gamete preparation medium, 37 °C, 5% CO_2_/95% air). Permeabilization was accomplished as described [11]. Briefly, washed spermatozoa were resuspended in cold PBS containing 0.4 U/ml SLO for 15 min at 4 °C. Cells were washed once with PBS, resuspended in ice-cold sucrose buffer (250 mM sucrose, 0.5 mM EGTA, 20 mM Hepes-K [pH 7]) containing 2 mM DTT. Sperm were incubated and spotted on eight-well slides, air-dried, and fixed/permeabilized in ice-cold methanol for 30 s. Acrosomal status was evaluated by staining with FITC-coupled PSA according to [44]. At least 200 cells were scored using a Nikon (Tokyo, Japan) microscope equipped with epifluorescence optics. Negative (no stimulation) and positive (10 μM free Ca^2+^) controls were included in all experiments. For each experiment, the data were normalized by subtracting the number of reacted spermatozoa in the negative control (range 18%–30%) from all values, and expressing the resulting values as a percentage of the acrosome reaction observed in the positive control (range 30%–50%).

### Indirect immunofluorescence in SLO-permeabilized sperm

Sperm were permeabilized with SLO and treated as indicated in the figure legends. For syntaxin1A immunofluorescence, sperm were spotted on round cover slips and fixed/permeabilized in 2% paraformaldehyde–0.1% Triton X-100 in PBS for 10 min at room temperature. For VAMP2 immunofluorescence, 20 μg/ml anti-VAMP2 antibody was added to the sperm suspension and incubated for 10 min at 37 °C before spotting. After fixation, sperm were incubated in 50 mM glycine-PBS for 30 min at 20 °C and 1 h in 5% BSA-PBS containing 0.4% polyvinylpyrrolidone (PVP) (40,000 average MW). Cells were labeled with an anti-syntaxin antibody (overnight at 4 °C, 1/50 whole serum in 3% BSA-PBS/PVP), followed by a TRITC-labeled anti-rabbit IgG (1 h at 20 °C, 10 μg/ml in 0.5% BSA-PBS/PVP). TRITC-labeled anti mouse IgG was used when anti-VAMP2 was the primary antibody. Cover slips were washed (3×) with PBS/PVP between incubations. Finally, cells were incubated 1 min in cold methanol and stained with Hoechst 33258 (20 min at 20 °C, 1 μg/ml in PBS) followed by FITC-PSA (30 min at 20 °C, 50 μg/ml in PBS), and washed with distilled water 20 min at 4 °C. Cover slips were mounted in Gelvatol, and examined with an Eclipse TE3000 Nikon microscope equipped with a Plan Apo 60×/1.40 oil objective and a Hamamatsu (Bridgewater, New Jersey, United States) Orca 100 camera operated with MetaMorph 6.1 software (Universal Imaging, Downingtown, Pennsylvania, United States). Background was subtracted and brightness/contrast were adjusted to render an all-or-nothing labeling pattern using Jasc Paint Shop Pro 6.02 (Jasc Software, http://www.corel.com). The presence of immunostaining in the acrosomal region was evaluated in at least 200 cells in three independent experiments. Data were normalized with respect to the percentage of positive cells observed in untreated samples (range 30%–70%).

### Statistical analysis

Data were evaluated using one-way ANOVA. The Tukey-Kramer post hoc test was used for pairwise comparisons. The results are listed in Tables S1–S8, which correspond to data depicted in Figures S1, 1, 2, 3, 4, 6, 7, and 8, respectively. Only significant differences (*p* < 0.05) are discussed in the text.

## Supporting Information

Figure S1 SNAREs Are Assembled in Neurotoxin-Resistant Complexes in Non-Permeabilized Resting Spermatozoa Spermatozoa maintained in gamete preparation medium were treated at 37 °C for 15 min with 150 nM BoNT/A (holotoxin) or 25 nM BoNT/F (holotoxin). Next, 25 μM TPEN (a zinc chelator that inactivates neurotoxins) was added, AE was activated by adding 10 μM A23187, and the incubation continued for an additional 15 min (black bars). Sperm were then fixed and AE was measured as described in Materials and Methods. Several controls are included (grey bars): background AE in the absence of any stimulation (control); AE stimulated by 10 μM A23187 (Iono); lack of TPEN effect on exocytosis (TPEN→Iono); inhibitory effect of the neurotoxins on exocytosis (neurotoxin→Iono); and block of neurotoxin activity by TPEN (TPEN→neurotoxin→ Iono). The data were normalized as described in Materials and Methods (mean ± standard error of the mean [SEM]). Statistical analysis is provided in Table S1.
(409 KB JPG).

Table S1 Accessory Data and Statistical Analysis for Figure S1
(38 KB DOC).

Table S2 Accessory Data and Statistical Analysis for Figure 1
(50 KB DOC).

Table S3 Accessory Data and Statistical Analysis for Figure 2A and 2C (62 KB DOC).

Table S4 Accessory Data and Statistical Analysis for Figure 3
(39 KB DOC).

Table S5 Accessory Data and Statistical Analysis for Figure 4B (37 KB DOC).

Table S6 Accessory Data and Statistical Analysis for Figure 6
(51 KB DOC).

Table S7 Accessory Data and Statistical Analysis for Figure 7
(72 KB DOC).

Table S8 Accessory Data and Statistical Analysis for Figure 8
(36 KB DOC).

## Abbreviations

AE: acrosomal exocytosis
BoNT: botulinum neurotoxin
EA: E230A
NP-EGTA-AM: *O*-nitrophenyl EGTA–acetoxymethyl ester
FITC: fluorescein isothiocyanate
PSA: *Pisum sativum* agglutinin
PVP: polyvinylpyrrolidone
SEM: standard error of the mean
SLO: streptolysin-O
SNAP25: synaptosome-associated protein of 25 kD
TeTx: tetanus toxin
TPEN: N,N,N′,N′-tetrakis (2-pyridymethyl) ethylenediamine
VAMP: vesicle-associated membrane protein
