# Supplementary material for: Dynamics of SNARE Assembly and Disassembly during Sperm Acrosomal Exocytosis
Source: PLoS Biol. 2005 Sep 6;3(10):e323. doi: 10.1371/journal.pbio.0030323 (PMC1197286; doi:10.1371/journal.pbio.0030323)
Supplement: Supplementary file 3 [file pbio.0030323.st003.doc]

Table S3

|  | **Mean** | **S.E.M.** | **S.D.** | **N** | **ANOVA** |
| --- | --- | --- | --- | --- | --- |
| control | 20.1 | 2.1 | 8.6 | 17 |  |
| Ca^2+^ | 11.6 | 0.8 | 3.1 | 17 |  |
|  |  |  |  |  |  |
| TPENCa^2+^ | 97.1 | 12.3 | 32.5 | 7 | b |
| BoNT/ECa^2+^ | 3.9 | 10.0 | 17.2 | 3 | a |
| TPENBoNT/ECa^2+^ | 119.2 | 28.3 | 49.0 | 3 | b |
| BoNT/ETPENCa^2+^ | 83.6 | 17.7 | 30.6 | 3 | b |
|  |  |  |  |  |  |
| BoNT/BCa^2+^ | 7.2 | 7.1 | 14.2 | 4 | a |
| TPENBoNT/BCa^2+^ | 90.0 | 10.0 | 17.3 | 3 | b |
| BoNT/BTPENCa^2+^ | 82.2 | 7.1 | 14.3 | 4 | b |
|  |  |  |  |  |  |
| TeTxCa^2+^ | 7.2 | 13.8 | 23.9 | 3 | a |
| TPENTeTxCa^2+^ | 103.0 | 9.1 | 15.8 | 3 | b |
| TeTxTPENCa^2+^ | 97.8 | 7.8 | 13.5 | 3 | b |
|  |  |  |  |  |  |
| TeTx+TPENNSF/αSCa^2+^ | 92.3 | 7.7 | 10.9 | 2 | b |
| NSF/αS+TeTx TPENCa^2+^ | 17.1 | 6.0 | 8.5 | 2 | a |
|  |  |  |  |  |  |
| Rab3A | 100.4 | 6.1 | 10.6 | 3 | b |
| TeTx/TPENNSF/αSRab3A | 97.2 | 2.8 | 4.8 | 3 | b |
| NSF/αS+TeTxTPENRab3A | 8.7 | 10.2 | 17.6 | 3 | a |

Row “control”: is the actual percentage of reacted cells in unstimulated sperm. It includes all the experiments used in Fig.2A and 2C.

Row “Ca^2+^”: is the responsiveness (i.e., % of reacted sperm upon stimulation with 10 μM free calcium - % of reacted cells in unstimulated sperm) observed in all the experiments used in Fig. 2A and 2C.

Other rows: data normalized as described in Materials and Methods [(% of reacted sperm under each condition-% of reacted cells in unstimulated sperm)/ responsiveness]*100. The last three rows represent incubation conditions identical to the previous two, except that Rab 3A was used instead of Ca^2+^. Notice the similar outcome in response to the two inducers. These data are not included in Figure 2.

Columns: show the mean, standard error of the mean, standard deviation, number of experiments and statistical analysis. The conditions were compared by one way ANOVA on normalized values. An independent ANOVA analysis was carried out for group of conditions separated by empty rows. The mean of conditions that share a letter do not differ significantly for P≤0.05.
