# Supplementary material for: Dynamics of SNARE Assembly and Disassembly during Sperm Acrosomal Exocytosis
Source: PLoS Biol. 2005 Sep 6;3(10):e323. doi: 10.1371/journal.pbio.0030323 (PMC1197286; doi:10.1371/journal.pbio.0030323)
Supplement: Supplementary file 6 [file pbio.0030323.st006.doc]

# Table S6

|  | **Mean** | **S.E.M.** | **S.D.** | **N** | **ANOVA** |
| --- | --- | --- | --- | --- | --- |
| control | 22.4 | 1.4 | 5.3 | 14 |  |
| Ca^2+^ | 11.9 | 0.8 | 2.9 | 14 |  |
| NPCa^2+^dark | 10.5 | 19.5 | 27.6 | 2 | a |
| NPCa^2+^h | 119.5 | 10.5 | 14.8 | 2 | b |
| NPBoNT/ECa^2+^h | 11.6 | 24.0 | 48.1 | 4 | a |
| NP Ca^2+^BoNT/Eh | 2.9 | 6.2 | 12.3 | 4 | a |
|  |  |  |  |  |  |
| Rab3A | 94.8 | 3.6 | 8.0 | 5 | b |
| NPRab3Adark | 3.2 | 12.1 | 20.9 | 3 | a |
| NPRab3Ah | 103.0 | 3.0 | 5.2 | 3 | b |
| NPBoNT/ERab3Ah | -7.4 | 11.5 | 19.9 | 3 | a |
| NPRab3ABoNT/Eh | 12.1 | 9.5 | 16.4 | 3 | a |
|  |  |  |  |  |  |
| NP Ca^2+^dark | 10.2 | 6.5 | 11.2 | 3 | a |
| NP Ca^2+^h | 103.7 | 3.7 | 6.4 | 3 | b |
| NPBoNT/C Ca^2+^h | 18.6 | 9.4 | 16.4 | 3 | a |
| NP Ca^2+^BoNT/Ch | 23.2 | 4.1 | 7.1 | 3 | a |

Row “control”: is the actual percentage of reacted cells in unstimulated sperm. It includes all the experiments used in Fig.6.

Row “Ca^2+^”: is the responsiveness (i.e., % of reacted sperm upon stimulation with 10 μM free calcium - % of reacted cells in unstimulated sperm) observed in all the experiments used in Fig. 6.

Other rows: data normalized as described in Materials and Methods [(% of reacted sperm under each condition-% of reacted cells in unstimulated sperm)/ responsiveness]*100.

Columns: show the mean, standard error of the mean, standard deviation, number of experiments and statistical analysis. The conditions were compared by one way ANOVA on normalized values. An independent ANOVA analysis was carried out for group of conditions separated by empty rows. The mean of conditions that share a letter do not differ significantly for P≤0.05.
