# Supplementary material for: Dynamics of SNARE Assembly and Disassembly during Sperm Acrosomal Exocytosis
Source: PLoS Biol. 2005 Sep 6;3(10):e323. doi: 10.1371/journal.pbio.0030323 (PMC1197286; doi:10.1371/journal.pbio.0030323)
Supplement: Supplementary file 8 [file pbio.0030323.st008.doc]

Table S8

|  | **Mean** | **S.E.M.** | **S.D.** | **N** | **ANOVA** |
| --- | --- | --- | --- | --- | --- |
| control | 46.7 | 7.9 | 13.6 | 3 |  |
|  |  |  |  |  |  |
| B-AMBoNT/B | 98.4 | 6.7 | 11.5 | 3 | b |
| B-AMBoNT/B Ca^2+^ | 49.0 | 3.8 | 5.4 | 2 | a |
| B-AM Ca^2+^BoNT/B | 45.7 | 1.0 | 1.7 | 3 | a |
| B-AMTeTx | 95.8 | 6.2 | 10.8 | 3 | b |
| B-AMTeTx Ca^2+^ | 46.0 | 5.6 | 9.8 | 3 | a |
| B-AM Ca^2+^TeTx | 96.7 | 4.1 | 7.1 | 3 | b |

Row “control”: is the actual percentage of sperm immunostained with an anti-VAMP2 antibody. It includes all the experiments used in Fig. 8.

Other rows: data normalized as described in Materials and Methods (% of immunostained sperm under each condition / % of immunostained sperm in unstimulated sperm) *100.

Columns: show the mean, standard error of the mean, standard deviation, number of experiments and statistical analysis. The conditions were compared by one way ANOVA on normalized values. The mean of conditions that share a letter do not differ significantly for P≤0.05.
